# Major Depressive Disorder in Youth: A Meta-Analysis of Functional Magnetic Resonance Imaging Studies

**DOI:** 10.1192/j.eurpsy.2023.512

**Published:** 2023-07-19

**Authors:** G. Zamora, C. Baten, A. M. Klassen, J. H. Shepherd, E. Pritchard, S. Saravia, Z. Ali, J. Jordan, S. K. Kahlon, G. Maly, M. Duran, S. L. Santos, A. F. Nimarko, D. W. Hedges, J. P. Hamilton, I. H. Gotlib, M. D. Sacchet, C. H. Miller

**Affiliations:** ^1^Psychology, California State University, Fresno; ^2^Psychology, Stanford University, Stanford; ^3^Psychology, Brigham Young University, Provo, United States; ^4^Biomedical and Clinical Sciences, Linköping University, Linköping, Sweden; ^5^Meditation Research Program, Department of Psychiatry, Massachusetts General Hospital, Harvard Medical School, Boston, United States

## Abstract

**Introduction:**

Major depressive disorder (MDD) is a highly prevalent mental illness that frequently originates in early development and is pervasive during adolescence. Despite its high prevalence and early age of onset, our understanding of the potentially unique neural basis of MDD in this age group is still not well understood, and the existing primary literature on the topic includes many new and divergent results. This limited understanding of MDD in youth presents a critical need to further investigate its neural basis in youth and presents an opportunity to also improve clinical treatments that target its neural abnormalities.

**Objectives:**

The present study aims to advance our understanding of the neural basis of MDD in youth by identifying abnormal functional activation in various brain regions compared with healthy controls.

**Methods:**

We conducted a meta-analysis of functional magnetic resonance imaging (fMRI) studies of MDD by using a well-established method, multilevel kernel density analysis (MKDA) with ensemble thresholding, to quantitatively combine all existing whole-brain fMRI studies of MDD in youth compared with healthy controls. This method involves a voxel-wise, whole-brain approach, that compares neural activation of patients with MDD to age-matched healthy controls across variations of task-based conditions, which we subcategorize into affective processing, executive functioning, positive valence, negative valence, and symptom provocation tasks.

**Results:**

Youth with MDD exhibited statistically significant (p<0.05; FWE-corrected) hyperactivation and hypoactivation in multiple brain regions compared with age-matched healthy controls. These results include significant effects that are stable across various tasks as well as some that appear to depend on task conditions.

**Conclusions:**

This study strengthens our understanding of the neural basis of MDD in youth and may also be used to help identify possible similarities and differences between youth and adults with depression. It may also help inform the development of new treatment interventions and tools for predicting unique treatment responses in youth with depression.

**Disclosure of Interest:**

None Declared

